# Advancing prevention and screening in younger adults living with low income: development, piloting and acceptability/appropriateness evaluation of A BETTER Life

**DOI:** 10.1186/s40814-025-01754-x

**Published:** 2025-12-17

**Authors:** Aisha K. Lofters, Kimberly Devotta, Tutsirai Makuwaza, Kimberly Lepine, Kris Aubrey-Bassler, Peter D. Donnelly, Carolina Fernandes, Eva Grunfeld, Jill Konkin, Donna P. Manca, Candace Nykiforuk, Lawrence Paszat, Andrew Pinto, Linda Rabeneck, Ambreen Sayani, Peter Selby, Nicolette Sopcak, Becky Wall, Mary Ann O’Brien

**Affiliations:** 1 Women’s College Hospital, Toronto, ON Canada; 2 Department of Family and Community Medicine, Faculty of Medicine, University of Toronto, Toronto, ON Canada; 3 Durham Community Health Centre, Oshawa, ON Canada; 4 Primary Healthcare Research Unit, Memorial University of Newfoundland, St. John’s, NL Canada; 5 University of St, Andrews, St. Andrews, Scotland; 6 Department of Family Medicine, University of Alberta, Edmonton, AB Canada; 7 Ontario Institute for Cancer Research, Toronto, ON Canada; 8 School of Public Health, University of Alberta, Edmonton, AB Canada; 9 Sunnybrook Research Institute, Sunnybrook Health Sciences Centre, Toronto, ON Canada; 10 Upstream Lab, MAP/Centre for Urban Health Solutions, Li Ka Shing Knowledge Institute, Unity Health Toronto, Toronto, ON Canada; 11 Institute of Health Policy, Management and Evaluation, University of Toronto, Toronto, ON Canada; 12 Department of Family and Community Medicine, St. Michael’s Hospital, Toronto, ON Canada; 13 Centre for Addiction and Mental Health, Toronto, ON Canada; 14 Durham Region Health Department, Whitby, ON Canada

**Keywords:** Prevention, Screening, Health promotion, Social determinants of health

## Abstract

**Background:**

In the original BETTER (Building on Existing Tools to Improve Chronic Disease Prevention and Screening in Primary Care) intervention, a “Prevention Practitioner” meets with a participant aged 40–65 years to improve their uptake of prevention activities (e.g. cancer screening, physical activity). The BETTER intervention was found to be effective in a randomised control trial. We adapted BETTER to focus on a younger age group (adults aged 18–39 years) living with low income, a group known to have a higher prevalence of preventable cancers and chronic diseases than their higher-income peers. Here, we describe the development, piloting, and qualitative evaluation of the acceptability of the adapted BETTER intervention (“BETTER Life”) to inform future large-scale implementation research.

**Methods:**

To support adaptation of BETTER, we interviewed community residents from low-income areas in Durham Region, Ontario, Canada and healthcare program service providers across Canada who had knowledge about preventive care. We developed an adapted intervention, BETTER Life, and piloted it at the Durham Community Health Centre to understand acceptability and appropriateness. Pilot participants were contacted a minimum of 2 weeks afterward to complete a semi-structured interview and share their experiences with the intervention and preventive care.

**Results:**

We conducted 22 adaptation interviews with 10 community residents and 12 healthcare service providers, 6 interviews with pilot participants (of 8), and a focus group with the two Prevention Practitioners. We found that participants felt that poverty contributes to poor health, including mental health; health education and interventions are often missing, unknown, or difficult to access in low-income communities; and that social networks are important for health. As a direct response to these issues, BETTER Life was seen as a unique, comprehensive program in the community that helps people set goals and reinforce healthy behaviours. However, many different strategies may be required to encourage engagement in the BETTER Life program.

**Conclusions:**

We developed BETTER Life by adapting the original BETTER to focus on adults aged 18–39 years living with low income, piloted it, and evaluated its acceptability and appropriateness. Although BETTER Life was seen as an important program, recruitment for the larger-scale study will be challenging as young adults struggle with competing life priorities and the social determinants of health.

**Supplementary Information:**

The online version contains supplementary material available at 10.1186/s40814-025-01754-x.

## Key messages regarding feasibility

### What uncertainties existed regarding the feasibility?

It was unclear if a program like BETTER, which involves a lengthy health-focused 1:1 visit, would be acceptable to a younger age group.

### What are the key feasibility findings?

BETTER Life was seen as an important and unique program that was acceptable and appropriate to participants. However, recruitment was very challenging.

### What are the implications of the feasibility findings for the design of the main study?

Recruitment for the larger-scale study will be challenging as young adults struggle with competing life priorities and the social determinants of health.

## Background

Canada, like many countries around the world, is currently facing an unprecedented rise in the prevalence of chronic diseases and cancers, many of which are preventable [1, 2]. Approximately 9% of Canadians were living with diagnosed diabetes in 2016–2019 [3]. In the same time period, nearly one in four Canadian adults had hypertension [4]. In addition, an estimated one in two Canadians will develop cancer in their lifetime, and one in four will die from cancer [5].

Primary prevention is the most effective way to avoid these chronic conditions from developing in the first place, while screening and secondary prevention can minimise their effect after development [6]. Lifestyle factors play a significant role in the development of many chronic conditions and cancers, and therefore addressing these is an essential component of primary prevention. For example, certain risk factors, in particular smoking, physical inactivity, and poor nutrition, account for approximately 25% of all direct medical costs [7]. High level evidence supports lifestyle modification and screening actions as leading to improved outcomes; however, healthcare systems are often unable to adequately support providers and patients to address and make these changes [8, 9].

Tailored interventions that use a combination of strategies have been shown to be the most effective way to implement care [10–13]. Accordingly, the BETTER (Building on Existing Tools to Improve Chronic Disease Prevention and Screening in Primary Care) approach was developed [8]. In the BETTER approach, the Prevention Practitioner—a new role undertaken by health professionals within primary care practices—meets in a one-on-one visit with participants aged 40–65 years to improve their uptake of prevention activities. The BETTER intervention was designed to motivate participants to undertake preventive actions (e.g. cancer screening, physical activity) that they are not currently undertaking, and was found to be effective in a randomised control trial [8]. At follow-up, participants in the immediate intervention arm met 64.5% of actions for which they were eligible versus 42.1% in the wait-list arm (rate ratio 1.53 [95% confidence interval 1.22–1.84]) [8]. Adaptations to BETTER have included public health nurses as Prevention Practitioners [14], and addressing the needs of cancer survivors [15].

The age range of 40 to 65 years was chosen for the original BETTER because most evidence-based tests for chronic disease are relevant to this age range in Canada (e.g. breast and colorectal cancer screening, glucose and lipid testing), but it may be more effective to address chronic disease prevention through lifestyle factors at younger ages. Among Canadians aged 18–34 years and 35–49 years, data from Statistics Canada indicated that in 2022 the prevalence of obesity was 24.6% and 33.2% respectively, heavy drinking 27.2% and 24.7%, and cigarette smoking 10.7% and 14.0% [16]. Reasons to focus on a younger age group are even more compelling for people living with low income: Canadians living in the lowest socioeconomic group are not only more likely to develop multiple chronic conditions, but the onset of chronic conditions is also likely to be significantly earlier [17]. Intervening earlier to reduce the accumulation of risk across the lifecourse is more likely to be effective at reducing chronic disease morbidity and mortality [18]. However, it is unclear if an intervention such as BETTER would be acceptable to, and deemed appropriate for, a younger age group.

We adapted the BETTER intervention to focus on a younger population of adults aged 18–39 years living with low income, a group known to have earlier mortality due to, and higher prevalence of, preventable cancers and chronic diseases than their higher-income peers. In this paper, we describe the (i) development, (ii) piloting, and (iii) qualitative evaluation of the acceptability and appropriateness of the adapted BETTER, hereafter referred to as BETTER Life, to inform future large-scale implementation research.

## Methods

### Ethics

This study received approval from the Research Ethics Boards at Women’s College Hospital (REB# 2020–0096-E) and the University of Toronto (REB# 00040912), and the Newfoundland and Labrador Health Research Ethics Board (Ref # 2021.011).

#### Original BETTER intervention

In the original BETTER intervention, Prevention Practitioners underwent a 2-day training curriculum that included review of relevant evidence-based guidelines relevant to the 40–65-year age group (e.g. cancer screening guidelines, recommended lifestyle modifications), principles of motivational interviewing, and shared decision-making in the context of small group discussions, cases, and role-playing [8, 19, 20]. This training allowed them to then hold one-on-one prevention visits with patients who had completed a detailed health survey prior to the visit. By reviewing survey results and any available medical records beforehand, the Prevention Practitioner focused on three core components during the visits:

i. Providing personalised information on prevention and screening in the form of a “bubble diagram” that the patient could take home with them
ii. Helping the patient to set specific, measurable, achievable, relevant, time-bound (S.M.A.R.T.) [21] health goals
iii. Providing the patient with a prevention prescription that could also be shared with their primary care provider.

### Development of BETTER Life intervention (adaptation of BETTER)

The ADAPT-ITT (Assessment, Decision, Adaptation, Production, Topical experts, Integration, Training, Testing) model is designed to facilitate the adaptation of evidence-based interventions in novel situations [22]. We used the ADAPT-ITT model [22] to guide the adaptation of the original BETTER intervention for the development, piloting, and evaluation of the BETTER Life intervention (Table 1). Specifically, we aimed to use evidence-based recommendations that were more relevant to this age group and were the basis of the prevention visit, and to adapt the health survey, bubble diagram, and prevention prescription to be more appropriate for this new target population.

**Table 1:** Description of how research team conducted steps in ADAPT-ITT* framework to apply to BETTER Life

Phase	Methodological decisions
Assessment	We conducted a literature review of evidence-based guidelines to identify relevant prevention and screening actions for the 18–39 years age group to include in BETTER Life. We also obtained input from public health and primary care experts across multiple provinces. This approach is described in detail elsewhere [23], and led to 42 recommendations that were at the core of BETTER Life
Decision	Using results from the literature review and expert input, the research team made initial decisions about questions to remove and add to the existing health survey, and necessary adaptations required to the various BETTER tools to reflect the younger age range, e.g. incorporating identifying substance use into survey and tools
Administration	We also held interviews with the target population and with healthcare service providers to understand barriers and facilitators (individual, interpersonal, and structural) to preventive care and lifestyle modification and explore elements for adaptation to BETTER Life
Production	We produced a further refined version of BETTER Life, including creating a training curriculum for Prevention Practitioners, adapted from the original BETTER training curriculum
Topical experts	Study team, clinicians and administrators in Ontario, Alberta, and Newfoundland, current Prevention Practitioners, and community residents provided input and feedback at each phase
Integration	We created a final adapted version of BETTER Life that integrates all findings
Training	We trained two Prevention Practitioners for the pilot, who were existing health promoters at a non-profit community-based organization providing primary care and health promotion services to marginalized populations. The Prevention Practitioners were trained on BETTER Life, including the preventive care recommendations for this age group and the adapted tools to be used during the visit. The training was led by a public health nurse with previous experience as a Prevention Practitioner
Testing	Acceptability testing was conducted and is described herein

^*^Source: Wingood GM, DiClemente RJ. The ADAPT-ITT model: a novel method of adapting evidence-based HIV interventions. J Acquir Immune Defic Symdr. 2008 Mar 1;47 Suppl 1:S40-6. https://doi.org/10.1097/QAI.0b013e3181605df1

To support the adaptation of BETTER for a younger age group, we first conducted an evidence review of high-quality guidelines to identify and assess prevention and screening actions for risk factors amenable to change for the 18–39-year age group. The process and results of this evidence review are presented elsewhere [23]. Briefly, we conducted a critical review of clinical practice guidelines on prevention and screening, and developed a list of 42 recommendations for 18–39-year-olds in areas such as diabetes, obesity, social determinants of health, and substance use. These recommendations were then incorporated into an adapted training curriculum and an adapted toolkit for Prevention Practitioners for use in BETTER Life.

To support adaptation, we also sought input through qualitative interviews with community residents from low-income areas in Durham Region, Ontario, Canada where we planned to conduct the piloting of BETTER Life, and with healthcare program service providers (administrators and clinicians) who had knowledge about preventive care in the provinces of Alberta, Ontario, and Newfoundland and Labrador, where BETTER has previously been implemented. The eligibility criteria for community resident participants were that they had to be between the ages of 18 years and 39 years, living in Durham Region and able to speak conversational English. We used a purposeful sampling strategy [24, 25] to obtain a range of views, including those of younger adults (18–29 years) and slightly older adults (30 to 39 years). We reasoned that the views of younger adults could be different from those who were a little older and potentially in a different stage of life, for example, concerns about healthy food choices for their children. We also wanted to include people who identified as women, men or other, including non-binary individuals. In these adaptation interviews, we asked community residents about the health of their neighbourhoods, their personal health, and perceptions of the BETTER intervention. We also asked service providers about the health challenges faced by community residents and the need for the BETTER Life intervention. Interviews were audio-recorded and analysed by two team members. Community residents received a $25 gift card for their time. Adaptation interviews took place from October 2021 to March 2022, and findings were used to inform the development of the intervention, such as emphasising mental health during the visit, ensuring Prevention Practitioners were aware of available programs to which participants could be referred, ensuring Prevention Practitioners were non-judgmental and non-alarmist in their approach, and ensuring both virtual and in-person visit options were available to participants to meet their needs.

### Description of BETTER Life intervention

In the BETTER Life intervention, participants first completed a baseline survey with a member of the research team using REDCap [26] that asked them a series of questions, including screening tools, pertaining to their lifestyle and health. After survey completion, one of the two Prevention Practitioners working on the pilot contacted each participant to schedule an in-person or virtual one-on-one visit depending on the participant’s preference. During the visit, the Prevention Practitioner reviewed the responses to the baseline survey and used the three BETTER Life tools (see Appendix 1) to guide their conversation with the participant:

i. *Prevention prescription*—summarises the prevention visit, where the participant is and where they should be, any recommended referrals or tests, as well as take home messages for the participant.
ii. *Bubble diagram*—completed with the participant based on their responses to the survey, and used to guide the visit. Multiple versions of the bubble diagram were created, to reflect recommendations that vary with gender identity (e.g. cervical screening).
iii. *Goals setting worksheet*—guided the participant to create up to three S.M.A.R.T goals, with support from the Prevention Practitioner, based on the areas identified through the prevention prescription and bubble diagram.

At the end of the visit, participants were given copies of the bubble diagram and goals setting worksheet to take home as a reminder of what was discussed at the visit and the goals that were set. If the participant had an accessible primary care provider, the prevention prescription was shared with them to include in their medical record.

### Piloting of BETTER Life

The primary purpose of the BETTER Life pilot was to understand its acceptability to, and appropriateness for, both participants and Prevention Practitioners. Using the definition by Proctor et al. [27], we defined acceptability as the perception among implementation stakeholders that a given innovation is agreeable, palatable, or satisfactory, based on their direct experience, and appropriateness as the perceived fit or relevance of the innovation for a given setting, consumer, or problem. The pilot was based in Durham Region, an area east of the city of Toronto with a population of approximately 700,000 people, and a region in which BETTER had previously been implemented [14, 28]. There were two Prevention Practitioners for the pilot, who were existing health promoters at the Durham Community Health Centre (CHC), which is a non-profit community-based organisation providing primary care and health promotion services through interprofessional teams with a focus on health equity and serving marginalised populations. The Prevention Practitioners were trained on BETTER Life, including the preventive care recommendations for this age group and the adapted tools to be used during the visit. The training was led by a public health nurse with previous experience as a Prevention Practitioner [14] and extensive knowledge of the Durham Region. The two Prevention Practitioners also participated in role-playing and a mock visit with a member of the research team.

#### Sample size justification

In qualitative research, participant recruitment ideally continues until data/thematic saturation is reached, which typically (but not always) occurs after 6–12 interviews [29, 30]. Thus, we set a target sample size of 12 BETTER Life participants at this pilot stage, but with our main goal being data saturation.

### Participant recruitment

Participants in BETTER Life were recruited from the client base of the CHC, as well as people from the broader Durham community. We posted study flyers in public spaces that serve people living with low income and on community websites, and the Prevention Practitioners regularly presented the study to their clients and attendees at other relevant programs within the CHC. Our eligibility criteria were similar to the community residents engaged in the adaptation phase, where participants were between the ages of 18 years and 39 years at the time of participation, able to converse in English, and lived in Durham Region. We recruited BETTER Life participants from July to September 2023.

#### Qualitative evaluation of acceptability and appropriateness of BETTER Life

We conducted a qualitative evaluation in order to understand the acceptability and appropriateness of BETTER Life. BETTER Life participants who completed their prevention visits were contacted a minimum of 2 weeks after their visit with their Prevention Practitioner to complete a semi-structured interview. All BETTER Life participants were invited to participate in an evaluation interview. During these interviews, participants were asked about their views and opinions on any steps they had taken, or any care they had received, to prevent chronic diseases. They were also asked about their experience with the Prevention Practitioner visit. Specifically, they were asked about why they decided to participate, any difficulties arranging the visit, what they liked and did not like about the visit, their views on the tools used, their ability to work on goals set during the visit, whether they thought others would be interested in BETTER Life, and how people in their neighbourhood could be reached to learn about BETTER Life.

As well, in August 2023, the two BETTER Life Prevention Practitioners participated in a focus group to provide input on acceptability and appropriateness from their perspective. During the focus group, the two Prevention Practitioners were asked about the existing programs in the community, the fit of BETTER Life in the CHC, any changes made at the CHC to accommodate BETTER Life, any barriers and facilitators to implementation, any key influences, their role and experience as a Prevention Practitioner, the skills and abilities needed to be a Prevention Practitioner, their confidence in their skills and abilities, their views on their training, lessons learned, perceived benefits and disadvantages, and overall views on the implementation and impact of BETTER Life. All interviews and the focus group took place over the phone or using Zoom videoconferencing. The interviews were audio-recorded and transcribed verbatim for the purposes of analysis. BETTER Life participants received $25 gift cards for their participation.

### Data analysis

Our analysis of qualitative data was based on principles of grounded theory. Grounded theory is a well-known qualitative method suited to examining a particular phenomenon to understand interactions and processes [31], such as the implementation of BETTER Life within this new context of younger adults living with low income. We used principles of grounded theory in previous BETTER studies [32, 33] and found it appropriate to study participant and service provider views. We used the constant comparative method for data coding [31, 34]. Initially, two team members (TM and KD) coded three transcripts in each data collection phase using an editing style of coding [35]. From the codes identified during this process, a preliminary coding guide was developed and reviewed with team members who are experienced qualitative researchers. Subsequently, TM and KD coded the rest of the transcripts. Periodic meetings with qualitative research team members were held to review the codes, sort codes into categories, and identify main themes [31, 34]. We conducted cross-comparison analyses of the community resident, Prevention Practitioner, and service provider data sets. During the coding, team members created memos that documented emerging relationships among the codes and categories. We used data management and analysis software (NVivo 12, QSR International) to assist with the analysis. We created interview summaries and memos to document all major decisions [36]. Involvement of members of the research team during the analytic process ensured that identified themes were consistent with coded data [37]. Demographics were captured in an Excel Database during the adaptation interviews and through the REDCap baseline survey for the acceptability/appropriateness interviews and for the focus group with the two Prevention Practitioners.

## Results

We conducted 22 adaptation interviews with 10 community residents and 12 healthcare service providers. These interviews lasted between 35 and 120 min. Amongst the 10 community residents, 9 identified as women and one identified as non-binary. The average age was 32 years (range 23–40 years) with the majority of participants living in households with a combined income of CAD $20,000–$39,000 from all sources before taxes. Almost all participants interviewed reported they were already living with a chronic illness (e.g. diagnosed with diabetes, high blood pressure, anxiety), had interest in improving their health, and viewed the BETTER Life program positively. For the 12 service providers, 9 identified as women and three identified as men. They were primarily affiliated with community-based primary care clinics, service organisations, and government agencies. Service providers reported working in their respective roles an average of 8.8 years (range 1–36 years). Participant demographics are presented in Tables 2 and 3.

**Table 2:** Summary of demographics for community resident participants from the adaptation phase (*n* = 10)

Participant characteristics	Interview participants* N* (%)
Gender identity	
• Women	9 (90%)
• Gender non-conforming/non-binary	1(10%)
Age	
• Range	23–40 y
• Average	32 y
Marital status	
• Single or never married	4 (40%)
• Married	2 (20%)
• Common-law/living with a partner	4 (40%)
Race	
• White	4 (40%)
• Black	4 (40%)
• Indigenous	1 (10%)
• Mixed race	1 (10%)
Highest education level	
• Completed high school	1 (10%)
• Some community college or technical school	1 (10%)
• Some University	1 (10%)
• Completed bachelor’s degree	6 (60%)
• Graduate degree	1 (10%)
# of people in household	
• Range	2–7
• Average	3.3
# of people in household below 18 years old	
• Range	0–5
• Average	1.7
Combined household income (from all sources the previous year)	
• $20,000-$39,000	4 (40%)
• $40,000—$59,000	2 (20%)
• $60,000 or more	1 (10%)
• Prefer not to answer	3 (30%)
Difficulty making ends meet at the end of the month	
• Yes	5 (50%)
• No	4 (40%)
• Sometimes	1 (10%)
Country of birth	
• Canada	7 (70%)
• Other	3 (30%)

**Table 3:** Summary of demographics for healthcare administrator and clinician participants from the adaptation phase (*n* = 12)

Participant Characteristics	Interview participants* N* (%)
Gender	
• Female	9 (75%)
• Male	3 (25%)
Age	
• Range	34–63 years old
• Average	45 years old
Primary organization affiliation	
• Primary Care	4 (33%)
• Government	
- *Chronic disease prevention*	2 (17%)
- *Primary health care*	3 (25%)
• Service Organization	2 (17%)
• Academic/Health Services Researcher	1 (8%)
**Primary Role in Usual Work**	
• Administrator	6 (50%)
• Clinician	4 (33%)
• Program Coordination	1 (8%)
• Chronic Disease Consultant	1 (8%)
**Length of time in Role**	
• Range	1–36 y
• Average	9 y

For the pilot, nine people participated in the baseline survey. Of those, eight people went on to complete a Prevention Practitioner visit and we were able to interview six of those eight participants to understand acceptability and appropriateness. Interviews lasted between 27 and 86 min. The focus group with the two Prevention Practitioners was 87 min. Of the six participants that we interviewed, four identified as female, one identified as male, and one identified as non-binary. The average age was 31 years, with participants ranging from 20 to 38 years. Half of the participants were living with a spouse or common-law partner, and the other half reported being single. Two out of six of the participants reported having issues making ends meet at the end of the month, and five reported a household income lower than the Ontario average of $80,000 [38] (Table 4).

**Table 4:** Summary of demographics for BETTER Life participants

Community resident participant characteristics	Baseline survey (*n* = 9)	PP visit (*n* = 8)	Post-PP visit qualitative interview (*n* = 6)
	*n* (%)	*n *(%)	*n* (%)
Gender identity			
Female	6 (67%)	5 (63%)	4 (66%)
Male	1 (11%)	1 (13%)	1 (17%)
Additional options combined to protect anonymity of participants (including: transgender, genderqueer – neither exclusively male or female, pansexual, aromantic/asexual, greysexual, demisexual and self-described)	2 (22%)	2 (25%)	1 (17%)
Age			
Range	20y to 38y	20y to 38y	20y to 38y
Average	31y	31y	31y
Marital status			
Single or never married	5 (56%)	4 (50%)	3 (50%)
Married	1 (11%)	1 (13%)	1 (17%)
Common-law/living with a partner	3 (33%)	3 (38%)	2 (33%)
Ethnicity/Race (check all that apply)			
Indigenous	2 (20%)	2 (25%)	2 (29%)
African/Caribbean/Black (e.g. African, African Canadian, Afro-Caribbean, etc.)	2 (20%)	1 (11%)	0
European (e.g. British, French, German, Greek, Polish, Russian, Spanish, Swedish, Ukrainian etc., but not including Icelandic)	2 (20%)	2 (22%)	2 (29%)
North American (e.g. American, Canadian)	2 (20%)	2 (22%)	1 (14%)
South Asian (e.g. Bangladeshi, East Indian, Pakistani, Sri Lankan, etc.)	1 (10%)	1 (11%)	1 (14%)
West Asian (e.g. Afghan, Iranian, Kurdish, Lebanese, Turkish, etc.)	1 (10%)	1 (11%)	1 (14%)
Highest education level			
Completed high school	1 (11%)	1 (13%)	1 (17%)
Some community college or technical school	5 (56%)	4 (50%)	2 (33%)
Completed college, technical school or bachelor’s degree	3 (33%)	3 (38%)	3 (50%)
Household income before taxes (from all sources, previous year)			
Less than $10,000	2 (22%)	2 (25%)	2 (33%)
$10,000 to $19,999	2 (22%)	1 (13%)	0
$20,000 to $39,999	1 (11%)	1 (13%)	0
$40,000 to $59,999	1 (11%)	1 (13%)	1 (17%)
$60,000 to $79,999	2 (22%)	2 (25%)	2 (33%)
$80,000 to $99,999	1 (11%)	1 (13%)	1 (17%)
Difficulty making ends meet at the end of the month			
Yes	4 (44%)	3 (38%)	2 (33%)
No	5 (56%)	5 (63%)	4 (66%)
Country of birth			
Canada	7 (78%)	6 (75%)	4 (66%)
Other	1 (11%)	1 (13%)	1 (17%)
Did not answer	1 (11%)	1 (13%)	1 (17%)

Below we present a comparison of the key themes that emerged from the acceptability/appropriateness evaluation where we interviewed pilot participants who had engaged with the BETTER Life Prevention Practitioner visit, and what we initially heard in adaptation interviews.

### Theme 1: poverty contributes to poor health, including mental health

In the adaptation interviews, limited financial resources, which led to a prioritisation of short-term needs—aptly described as “the tyranny of the moment” by a community resident—were seen as significant barriers to younger adults’ capacity for engaging in activities that promote good health. Both service providers and community residents described many individuals as having minimum wage jobs, being employed in physically demanding shift work, having less money to buy nutritious food, and lacking access to health education or healthcare—all contributors to poorer physical health when compared to peers with greater financial resources.

Similarly, in the acceptability and appropriateness interviews, BETTER Life pilot participants discussed the challenges and impacts of living in poverty and how that contributed to poor health through such realities as food insecurity. Community residents described how poverty limits their access to healthy food:

Yeah, eating poorly, eating bad, food that is bad for you, because that's what's available and what's cheap, or what the food bank gives you. The food bank gives you a lot of stuff that's not very healthy, like a lot of bread and a lot of frozen junk and other junk food or food that's full of sugar, and people become obese. And then another way of becoming unhealthy.—BETTER Life pilot participant BL2303

So it's also a factor of money. Like I know it is for me, like I can't always eat the foods I would love to eat, because I just can't afford them all the time. So, when it comes to finances, it is a big one—BETTER Life pilot participant BL2302

Poverty and its impacts on mental health were also discussed by community residents in both adaptation and acceptability/appropriateness interviews. In adaptation interviews, both community residents and service providers reported mental health concerns as a priority for low-income younger adults. Community residents in the 18–39-year age range were viewed as being in relatively good physical health, but more often facing mental health challenges which could be associated with childhood trauma and/or the challenges of transitioning from adolescence to adulthood or even navigating parenthood in communities with limited resources (e.g. meeting spaces, safe walking trails, and grocery stores). Precarious mental health was seen as having been exacerbated by the coronavirus disease (COVID-19) pandemic, which further heightened feelings of anxiety, depression, and isolation for some individuals.

In this interview excerpt from a BETTER Life pilot participant, the individual talks about how friends and neighbours have challenging financial situations, and this leads to feelings of hopelessness.

For the most part, they're all stressed out and hopeless. And just trying to get through every day, one at a time, because the people around me, especially because I'm in [public] housing, there's people that like, you know, can't pay their bills, and they're just everyday figuring out what are they going to do? What are you going to do? Like I said, my friend's been applying and applying and applying. And then all of a sudden, the payments, that wasn't supposed to come out came out. And then she's got an NSF [no sufficient funds] fee, and then decide another thing. And next thing, you know, she's got, like, she's got negative dollars, and then somebody turned around and froze their card…You can’t afford to be poor, because that's charging for being poor. Your best isn't always good enough. And there's nothing all the time, there's nothing you can do about it. And that's why people start giving up.—BETTER Life pilot participant BL2303

### Theme 2: health education and interventions are often missing, unknown or difficult to access in low-income communities

In the adaptation interviews, participants described healthcare for this age group as reactive versus preventive, with a dearth of information-sharing about health resources and supports among peers:

I would say, like in my personal opinion …there's a lack of information sharing that goes on in how to obtain resources and to use those from a health promotion lens.—Community Resident 6

A service provider also highlighted the challenge young adults can face in finding appropriate programs:

Again, if you’re 18 years old and facing chronic disease yourself, it’s challenging to find those [community programs]. You might be moving - but you might not be moving - from the pediatric system into the adult system, so I think there are a lot of challenges that go along with navigating and finding the particular programs for yourself.—Service provider 8

Access and connection to primary care was specifically highlighted by service providers. One was concerned that the lack of available community programs meant BETTER Life would struggle to be successful without direct connection to primary care:

I think it would be not very advantageous to have it based in the community not connected to primary care. The evidence that we have for the benefit of that kind of model is when it's part of a team-based care approach, to have it be a standalone and not part of any team, I think would, it would be likely of limited benefit and could in fact be disruptive.—Service provider 11

Another was hopeful that BETTER Life could facilitate connections to primary care.

And I think that’s a really good opportunity for people to be connected within their community and can hopefully broaden the number of people that are able to access primary care in some capacity.—Service provider 8

Similarly, in the acceptability and appropriateness phase, BETTER Life participants discussed the availability, or lack thereof, of services and programs in the community. For example, one participant talked about programs that no longer exist:

And a lot of it comes to the fact that there’s, one, there’s a lot of young parents down here because there’s nothing for youth to do. I grew up in this area and we had camps in the parks. We had what [organization name] used to be when it was in the [location], there was a youth room you could go drop in at. There was easy access to these preventative programs and they’re not here anymore. So the youth are out on the streets. They’re getting into trouble. They’re just—not malicious; they’re bored. And there’s no supervision.—BETTER Life pilot participant BL2304

The same participant also discussed how current programs can be difficult to access:

I’ll use [organization name] as an example. A lot of the programming that happens at their buildings is phenomenal. And yes, we are in such close proximity to them. But in my neighbourhood it’s a 10, 15-minute walk. However, that is such a deterrent. And I’ve actually worked alongside a number of people who live in this neighbourhood and just trying—there’s a weekly meal program there—just trying to get them to come meet me there. They won’t do it unless transportation is provided.—BETTER Life pilot participant BL2304

### Theme 3: social networks are important for health

In the adaptation interviews, community residents viewed making social connections as important for younger adults.

Friendships, relationships [suggested topic for Prevention Practitioner visit], how to create them, how to hold onto them. You know. I think that that's another, like little pandemic that's happening within my age group. Not like momentous relationship but like friendship. Yeah, I find that a lot of people, especially right now—well there's the pandemic they're isolated—they—like the phone thing is kind of like messing people up.—Community Resident 9

Many pilot participants discussed the importance of social networks and how they interacted with others. Here, this participant emphasises the importance of social connections:

I don't see very many smokers. I see people outside walking their dogs. Not on their phones. I see people in open conversation with each other and not just quietly walking... When you’re able to, I think, talk to people and ask people for things I feel like you’re also able to take care of yourself better. – BETTER Life pilot participant BL2305

Another pilot participant discussed how important it is to surround yourself with people who will positively impact your mental health:

Surround yourself with good people, surround yourself with people who have a good mentality and can provide you with the help that you need. And it also factors down to the point where you need to have people who have, who you want to have the same mindset as. And then if you surround yourself with those people it gets you out of that habit of being like, “Oh, I can’t do it, I just can’t. I need the motivation.” It’s like no, if you’re around people who have the same mindset as you and you just really surround yourself with genuine good people, your mentality will be so much more better to where you’ll just start doing things on your own. People are very influential. Most don’t understand that but most do. And if you do surround yourself with very good people it will benefit you. - BETTER Life participant BL2307

### Theme 4: BETTER Life is a unique and comprehensive program in the community for younger adults that helps people set attainable goals and reinforce healthy behaviours

In adaptation interviews, service providers emphasised that BETTER Life would need to take a holistic approach toward health to engage this younger age group:

“…when you’re beginning with the younger cohort in that 18 to 39, it’s about establishing a really positive attitude towards life. It’s about establishing a really positive attitude towards your mental and your physical health… You want to establish a good attitude towards wellness, wellbeing, you know being engaged, taking part in activity, taking part in life because that’s what they want to do right? …”—Service provider 1

This aligned with findings from the pilot, where, overall, BETTER Life was viewed by both participants and Prevention Practitioners as acceptable and appropriate, because it was seen as a unique program that used a comprehensive approach to addressing many aspects of people’s lives that impact their health. Participants valued that it was a comprehensive program rather than disease-specific. As one of the Prevention Practitioners described:

….what the BETTER Life program does is it’s a specific program and I don't see or I'm not aware of anything else like that in the Durham area. And even speaking to other—like our clinical team when we were talking about people in our organization, as we were trying to promote it, they did seem—like, you know, no one had mentioned that there was anything else like this going on and they were very excited to refer people to it for the research component. And I thought it was super-beneficial to people and to this age group. So, yes, I don't think there’s any other.—Prevention Practitioner2

Community residents felt comfortable in their visit with the Prevention Practitioner. For some, the visits were a good opportunity to reinforce changes they were already making or thinking of making.

It's always like I said, it is nice to hear that you're on the right path. Because like there's those lows when you're progressing, right?—BETTER Life participant BL2302

The community health centre model was viewed as an appropriate and effective environment for the BETTER Life program. During the focus group, the Prevention Practitioners commented on how the BETTER Life program was very aligned with the existing programs and structures of the community health centre in which they were already working.

Yes, I think it completely makes sense because we do have ourselves, like our health promoters, like a whole team of health promoters that work interprofessionally with doctors and nurses and nurse practitioners, dietitians and social workers, and we're kind of like our area is on prevention, right? So, and we do have the ability to see people in the community and provide that service. And like I said, that's kind of our specialty as well is prevention. So, I definitely see it fitting within the Community Health Centre model.—Prevention Practitioner2

Positive views from participants about the BETTER Life tools that were used during the visit with the Prevention Practitioners contributed to acceptability. These tools were seen as highly relevant for the visit and setting goals.

[T]he template just really breaks it down for some people, and it just really helps them be like, “OK, I could improve on my exercise.” So it would just bump that up, and then there’s nutrition-wise, cutting back down on habits. And it’s also just a really good overall way to help someone navigate their goal.—BETTER Life participant BL2307

The goals setting worksheet was identified as particularly appropriate and acceptable because it was a hard-copy visual that also showed the goals to be measurable and attainable:

[T]hey were visual so you could follow the structure of your goal setting …they reminded me a lot of, like, smart goals. They’re very measurable and attainable. But yeah. I like that it was a visual somebody could take this home, hang it up on their fridge and follow it…I also—like, I do really appreciate the fact that they gave hard copies of the information too because it just—it validated how thoughtful this whole process has been of, OK, we recognise everybody might not have access to email copies or printing, so. And that was—they didn’t even vocalise that that was part of it or ask; they just automatically gave it…so it didn’t create any stigma so it was beautiful to see that approach being put into place first.—BETTER Life participant BL2304

### Theme 5: several strategies may be required to increase recruitment to the BETTER Life program

Recruitment was slow and at lower numbers than expected. Community residents discussed what could be done to engage more people to take part in BETTER Life. One community resident noted the potential impact of word-of-mouth and that hearing about the program from a peer that you trust could heighten perceived appropriateness:

Through people who care. So word of mouth and through promoting it through—like, how I heard of it. So I’ve heard it through [program] and through the people through there who care about other people.…I guess it’s how it’s presented. Like if I saw it presented in a way that was like, hey, you know, this is for this. And it’s straightforward. I think people like straightforward.—BETTER Life participant BL2305

Social media was also highlighted as a way to raise awareness among younger community residents:

Social media is the best way to reach the younger generation, but most kids my age are in the younger generation [who aren’t] socially active, but they’re out and about and just doing what they do.—BETTER Life participant BL2305

More community engagement and having a memorable presence were talked about by both community residents and the Prevention Practitioners as a way to build awareness and perceived appropriateness, and thus increase participation, for the local community:

I think just a lot more community engagement. And there are trusted persons within the community who you would need to build a relationship with and then those by kind of proximity, then you’ll start to build those relationships with people within the community. So things like Durham community health centre is a great partnership there. They’re here. They’re trusted. [name of group], the local community garden. Like, these are all places that are present in the community to start building those relationships… I think it’s great that data is being collected on the priority neighbourhoods here within Durham region. I’m just hopeful to see this data turn into community prevention programs that are, you know, noticeable and well attended.—BETTER Life participant BL2304

So, taking the posters to—you know, in the summer time we do a number of different community events where we go out into high-risk communities and, you know, we have activities and food and the Food Bank comes and different community organizations, and this is an opportunity for the Durham Community Health Centre to really promote their services..So we were able to do that, and that was a great way to be able to…to go out and to help us to promote the program.—Prevention Practitioner1

## Discussion

Adapted from the original BETTER approach and informed by a critical literature review [23] and interviews with community residents and service providers, the BETTER Life program was developed for people between the ages of 18 and 39 years living with low income, with a focus on integrating key chronic disease prevention and screening recommendations. These recommendations consider the age, sex, gender, health conditions, family history, and other lifestyle factors of participants, and were incorporated into a one-on-one prevention visit with a Prevention Practitioner. We piloted the BETTER Life intervention with 8 community residents and evaluated its acceptability and appropriateness with 6 of those participants. Our qualitative findings showed that participants viewed that poverty contributes to poor health, including mental health; health education and interventions are often missing, unknown, or difficult to access in low-income communities; and that social networks are important for health. As a direct response to these issues, BETTER Life was seen as a unique, acceptable, appropriate, and comprehensive program in the community for younger adults that helps people set goals and reinforce healthy behaviours. Participants were satisfied with the intervention, and it was seen as a good fit for the target population and local community setting. However, BETTER Life cannot completely fill the gap for much-needed community programming, including primary care, and many different strategies may be required to encourage engagement in the BETTER Life program.

As noted in our interviews, community residents would likely need to hear about the program in a variety of ways, from several trusted sources, over time, to make the commitment to participate [39]. Many younger adults may not inherently see the need for a program focused on prevention and screening, and different messaging around the focus and benefits of the program may need to be tried in order to increase interest. Although topics covered in BETTER Life included those relevant to this younger age group such as mental health, sexual health, and substance use, this focus may not have been clear in our recruitment approaches. As well, and as noted in interviews, many potential participants were likely dealing with competing life priorities related to the social determinants of health and mental health, which would by necessity be prioritised over preventive care [40–42]. The time required to commit to a survey followed by a prevention-focused visit simply may not have been feasible or important for some.

We found that poverty is a key driver in the lives of our target population, that health programs are lacking and difficult to access in low-income communities, and that social networks are important for health. Each of these themes can be informative for future participant recruitment. Future recruitment efforts may need to explicitly promote that Prevention Practitioners could enable connections to social services, mental health supports, and support groups that do exist, that Prevention Practitioners may be aware of health and other programs that the general public does not know about, and be able to provide navigation support, and that BETTER Life allows for a supportive connection with someone who is taking a person-centred approach. This messaging may be more compelling than a focus on prevention and screening.

For those who did participate, the comprehensive and personalised approach was appreciated and seen as beneficial to setting health goals and encouraging healthy behaviours. Previous BETTER versions have been similarly positively perceived. Among adults 40–65 years in primary care practices in Newfoundland, patients appreciated the visit with a Prevention Practitioner and wanted to receive sustained prevention and screening support [43]. In the BETTER WISE study, which included cancer survivors, participants highlighted the creation of a safe and trusting environment that empowered patients to set goals [44], and in BETTER HEALTH, where public health nurses were in the role of Prevention Practitioner and participants were living with low income, participants described feeling listened to and valued and that they had achieved at least one health goal despite daily stressors that reduced their capacity to make lifestyle changes [45]. In their review of the literature, Bonnie et al. found that for young adults in particular, health interventions that are multi-pronged, tailored to individual behaviours, and take into account social contexts are generally the most successful [46]. Notably, the authors highlight that there is limited evidence on what strategies may be most effective in engaging marginalised communities [46].

Future research will involve observational implementation research to assess uptake and effectiveness of BETTER Life. Previous BETTER studies have used a composite index that incorporates multiple prevention and screening actions for which each participant is eligible at baseline [8, 14, 15] to determine how many are achieved after receiving the intervention, and a similar index will be developed for BETTER Life. However, we will require intensive recruitment efforts, as noted above, to ensure a large enough sample size to assess effectiveness. Further research could also consider the contexts in which BETTER Life could be investigated (e.g. primary care, community).

A strength of this study was the positive perception of BETTER Life by both pilot participants and Prevention Practitioners. However, there are several limitations to note. First, we are uncertain that we achieved data saturation on all themes in evaluation interviews. As noted, we did not achieve our target sample size of 12 participants in the BETTER Life pilot, potentially influenced by the pandemic, and as a result had six interviewees. However, all six people spoke extensively about the influence of poverty on health. Second, it is possible that there was volunteer bias wherein the people who chose to participate were already motivated to improve their health and perhaps more likely to be housing and food secure. Finally, we were only able to offer the intervention in English, which may exclude some of those most in need of support.

## Conclusion

We developed BETTER Life by adapting the original BETTER intervention to focus on adults aged 18–39 years living with low income, piloted it, and evaluated its acceptability and appropriateness. Although BETTER Life was seen as a unique and useful program, recruitment for the larger-scale study will be a challenge as young adults struggle with competing life priorities, the social determinants of health, and mental health.

## Supplementary Information

Supplementary Material 1.

## Data Availability

The datasets used and/or analysed during the current study are available from the corresponding author on reasonable request.
